# Integrated analysis of ARHGAP6 potential function and prognostic value in acute myeloid leukemia

**DOI:** 10.1371/journal.pone.0333409

**Published:** 2025-10-07

**Authors:** Debin Yang, Yuanzhe Li, Ping Ma, Fankai Xiao

**Affiliations:** 1 Department of Pediatrics, Children’s Hospital Affiliated of Zhengzhou University, Zhengzhou, China; 2 Deparment of Hematology and Oncology, Children’s Hospital Affiliated to Zhengzhou University, Zhengzhou, China; 3 Oncology department, the First Affiliated Hospital of Zhengzhou University, Zhengzhou, Henan, China; Neyshabur University of Medical Sciences, IRAN, ISLAMIC REPUBLIC OF

## Abstract

**Background:**

Leukemia recurrence continues to be the primary cause of treatment failure, it is meaningful to find new biomarkers for its treatment. In this study, we aim to use cell line study to assess the expression and prognostic value of ARHGAP6 in acute myeloid leukemia.

**Methods:**

We applied two acute myeloid leukemia cell lines in the research. Expression level, proliferation assay and apoptosis assay for ARHGAP6 in those cell lines were involved for the study. Then, GEO, TCGA data and bioinformatics analysis were evaluated.

**Results:**

THP-1 and U937 cell lines both had higher expression levels of ARHGAP6 than control.

The cell proliferation of THP-1 and U937 transfected with ARHGAP6 siRNA was significantly reduced. Knock down the gene ARHGAP6 increases AML cell apoptosis. The overall survival (OS) and disease-free survival (DFS) was assessed against the expression of ARHGAP 6 using the KM plotter databases. High expression ARHGAP6 was associated poor OS and DFS in AML. Enrichment analysis suggested that ARHGAP6 mainly mediated the function of growth factor binding, immunoglobulin binding, mRNA binding. Involved in LCK proto-oncogene, Src family tyrosine kinase, tyrosine kinase non receptor 2, platelet derived growth factor receptor beta and Rho associated coiled-coil containing protein kinase 1.

**Conclusion:**

From cell lines-based functional assays to bioinformatic analysis, this study demonstrated that clinical potential of ARHGAP6 as a novel biomarker of AML.

## Introduction

An aberrant differential and a varied prognosis are the outcomes of acute myeloid leukemia (AML) [1], a hematological malignancy brought on by the early arrest of aberrant leukemia cell clones [2]. Leukemia recurrence continues to be the primary cause of treatment failure, despite the fact that more than half of AML patients achieve complete remission (CR) with induction chemotherapy, according to risk stratification-guided post-remission treatment methods [3]. Chemotherapy alone has a 40–50% likelihood of recurrence, whereas the prognosis for AML patients with t(8; 21) is better [4]. This emphasizes the need for new indicators that can more accurately guide treatment and evaluate prognosis.

By transforming the tiny G proteins RhoA and Cdc42 into their inactive GDP-bound forms, GTPase-activating proteins (RhoGAPs), which are expressed by genes belonging to the Rho GTPase Activating Protein (ARHGAP) family, negatively regulate Rho GTPases. Numerous cellular processes, such as cell cycle progression, survival, motility, polarity, adhesion, migration, and invasion, are significantly influenced by RhoGAPs [5–7]. ARHGAP family genes have been linked to treatment outcomes and have been demonstrated to be dysregulated in a variety of malignancies [8]. AML cell growth was decreased when ARHGAP43 (SH3 BP1) expression was down-regulated [9]. Patients with 13q14 and 11q22-23 deletions in chronic lymphocytic leukemia had lower survival rates when their ARHGAP20 expressions are higher [10]. ARHGAP4 may suppress DRAM1 in AML cells by interacting with p53. Additionally, DRAM1 knockdown corrects ARHGAP4 abnormalities in AML cells [11]. For follicular lymphoma, increased expression of ARHGAP24 is a substantial and independent adverse prognostic factor [12]. Prior research have demonstrated that ARHGAP26 expression in AML was substantially lower than in the control group [13].

For the treatment of AML, the discovery of new and trustworthy biomarkers is essential for precisely determining prognosis and creating therapy regimens. The roles of the ARHGAP family in AML have not been well studied by experts. The signaling transduction mediated by Rho guanosine triphosphatase hydrolase enzymes (GTPases) plays a pivotal role in the progression of human malignant diseases. Specifically, Rho GTPase-activating protein 6 (ARHGAP6) modulates actin polymerization, thereby facilitating tumor growth and metastasis [14]. ARHGAP 6 involved in several cancer progression [15,16], however, less report concerned its expression and function in AML We showed in this study that AML have overexpressed ARHGAP6. In AML cell lines, deletion of ARHGAP6 causes apoptosis and suppresses cell growth. Meanwhile, we conducted bioinformatics analysis of clinical characteristics and survival statistics associated with ARHGAP6 in AML. All of these results imply that the ARHGAP6 is essential for the development of AML and could be a viable therapeutic target for AML treatment.

## Methods

### Cell culture and transfection

We purchased the leukemia cells (THP-1 and U937) from the Procell Life Science Technology Co., Ltd. (Wuhan, China). THP-1 and U937 cells were cultivated at 37°C with 5% CO2 in RPMI 1640 (Pricella, PM150110) supplemented with fetal bovine serum (EXCELL.FSP500) and penicillin/streptomycin. Using Lipofectamine 3000 (Invitrogen, USA), the cells were transfected with the negative control (si NC) and ARHGAP6 siRNA. Table 1 lists the siRNA sequences that target ARHGAP6. To evaluate off-target impacts, cells underwent treatment with each siRNA at concentrations of 1 nM, 10 nM, and 25 nM, and were then contrasted with a non-targeting control siRNA via Affymetrix microarrays. Off-targets were characterized as transcripts exhibiting a twofold alteration in mRNA levels (RMA-normalized values) accompanied by a P-value of 0.05 or lower at any given dose. This criterion for off-targets permits the inclusion of both upregulated and downregulated transcripts, irrespective of whether they are directly targeted by the siRNA/RISC complex.

**Table 1:** SiRNA sequence.

	Sequence	GC%
SiRNA1	GUCCAUAGAAAUUCUUUGAAGCUUGAAAUUCUAGCUUAACAU	28.6%
SiRNA3	AUUUCUUUCUCUUUUGUCCAUGGACAAAAGAGAAAGAAAUCU	65.7%
SiRNAcontrol	UCUUUUAUUUCCAAAUGGGAGCCCAUUUGGAAAUAAAAGACA	60.0%

### Quantitative real-time polymerase chain reaction (qRT-PCR)

Total RNA from monocyte, THP-1, and U937 cell lines was extracted using the TRIzon reagent (CW0580S, Cwbio, China). 500ng RNA were retrotranscribed into cDNA using UEIris RT mix with DNase (US Everbright Inc, Jiangsu, China). 2 × Universal SYBR Green qPCR Supermix(US Everbright Inc, Jiangsu, China) was used to perform qRT-PCR. The PCR amplification conditions consisted of 45 cycles of 94°C for 10 min, 94°C for 10 s, and 60°C for 45 s each. The internal reference was GAPDH. Table 1 lists the primer sequences that were employed.

### Western blot

After lysing the samples in RIPA buffer (P0013B, Beyotime, China), they were denatured for 15 minutes at 100°C. The protein samples were transferred to polyvinylidene fluoride (PVDF) membranes after a 10% SDS-PAGE separation. The primary antibodies anti-ARHGAP6 antibody (1:2000, Invitrogen PA5–104106) and anti-beta-actin antibody (1:1000, CST 4967) were incubated on PVDF membranes for an extra hour after blocking them with a 5% skim milk powder solution. After then, secondary antibodies were left on for two more hours at room temperature. Protein band signals were through an electrochemiluminescent system using BCA Protein Assay Kit (CW0014S, Cwbio, China).

### Proliferation assay

Using the Cell Counting Kit-8 test (UElandy, China), proliferation was determined. Following EdU treatment in accordance with the manufacturer’s instructions (UElandy, Suzhou, China), cells were photographed using the EVOSTM Auto 2 imaging system (Thermo Fisher, USA) in randomly chosen fields. A microplate reader was used to determine each well’s O.D. 450 readings following two hours of incubation at 37°C.

### Apoptosis assay

As directed by the manufacturer, Annexin V/PI (propidium iodide) staining was carried out using the Annexin FITC/PI Apoptosis Detection Kit (Elabscience,China). In short, cells were cultured for 24 hours in a 6-well plate, after being extracted, the cells underwent two rounds of cold PBS washing. Additionally, in accordance with the manufacturer’s instructions, apoptotic cells were identified using an Annexin V-FITC apoptosis detection kit (Elabscience Biotechnology Co., Ltd.). Lastly, a flow cytometer (Pukang,China) was used to determine the proportions of cells at the early and late apoptotic stages.

### GEO, TCGA data and bioinformatics analysis

Tumor gene expression and methylations are made easier with the use of the UALCAN database. You can configure the parameters for data mining and filtering in this database. For the alteration analysis, the cBio Cancer Genomics Portal (http://cbioportal.org) is an open-access resource for interactive study of multidimensional cancer genomics data sets. For gene expression and overall survival analysis using tumor and normal samples from the TCGA and GTEx databases, the GEPIA (Gene Expression Profiling Interactive Analysis) web service has proven to be a useful and frequently mentioned resource. In order to illustrate the relationships between genes and pathway for ARHGAP6 in AML, the linkedomics enables flexible exploration of correlations between a molecular or clinical attribute of interest and all other characteristics. All websites can be found in S1 File.

## Results

### Expression level of ARHGAP6 in AML cell lines and monocyte

**Fig 1:**
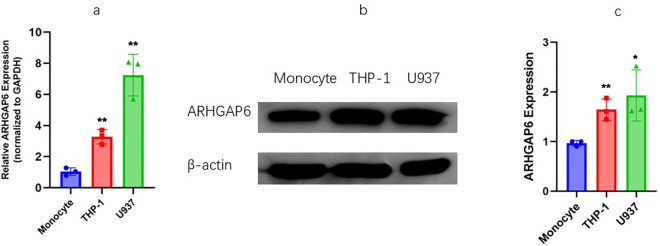
Expression of ARHGAP6 in AML cell lines. A qPCR assay to measure the expression levels of ARHGAP6in monocyte, THP-1 and U937 cells. B-C Western-blot to measure the expression levels of ARHGAP6 in monocyte, THP-1 and U937 cells and quantify the gray values.

We used PCR and WB to explore the expression of ARHGAP6 in monocyte, THP-1, and U937 cells in order to analyze the expression of ARHGAP6 in both leukemia cells and normal monocytes (Fig 1A–C). The raw image is in S1 Fig.The findings demonstrated that THP-1 and U937 cell lines both had higher expression levels of ARHGAP6 than control.

### ARHGAP6 promotes the growth of AML cell lines

**Fig 2:**
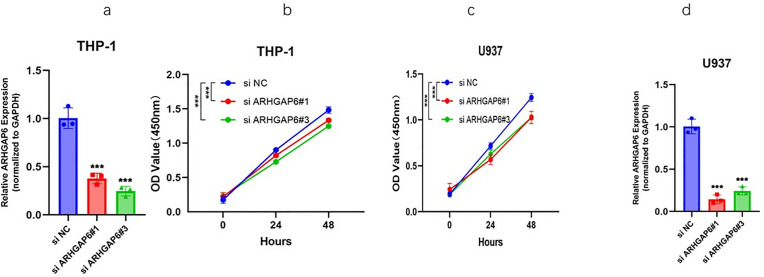
Proliferation assay of ARHGAP6 in AML cell lines. (a-b) Silencing ARHGAP6 in THP-1 cell line and assay of cell viability after inhibition (n = 3); (c-d)) Validation of the inhibition efficiency of ARHGAP6 in U937 cell line.

We created two short interfering RNAs targeting distinct ARHGAP targets, and we tested the effectiveness of the inhibition in THP-1 and U937 cell lines by q-RTPCR (Fig 2A, C). The siRNA knockdowns were also confirmed at the protein level using Western blot. The Western blot data in the S2 Fig to strengthen our validation. The CCK-8 assay was used to determine whether the silencing effect of ARHGAP6 inhibits the growth of human ARHGAP6 cells, THP-1 and U937. In comparison to the matching SiARHGAP6 and Negative control cells, the cell proliferation of THP-1 and U937 transfected with ARHGAP6 siRNA was significantly reduced, as illustrated in (Fig 2B, D). These findings imply that ARHGAP6 knockdown inhibits the growth of AML cells.

### ARHGAP6 silencing induces AML Cells to Apoptosis

**Fig 3:**
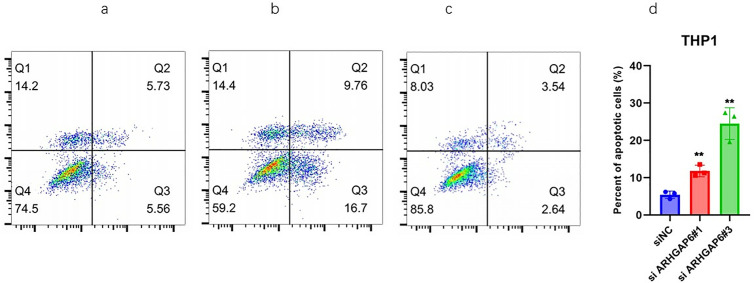
Knock down ARHGAP6 on AML THP1 cell death analysis. a Si1ARHGAP6 flow cytometry analysis b Si3ARHGAP6 flow cytometry analysis c Control group flow cytometry analysis d low expression of ARHGAP6 confers increase apoptotic activity.

**Fig 4:**
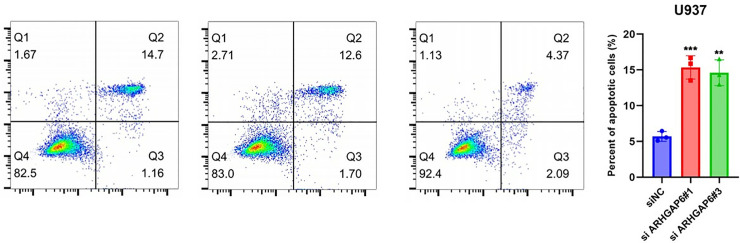
Knock down ARHGAP6 on AML U937 cell death analysis. a Si1ARHGAP6 flow cytometry analysis b Si3ARHGAP6 flow cytometry analysis c Control group flow cytometry analysis d low expression of ARHGAP6 confers increase apoptotic activity.

Annexin V-FITC/PI double-staining was used in flow cytometry (FCM) to evaluate apoptosis in THP-1 and U937 cells. Figs 3 and 4 displayed the rates of early apoptotic cells (located in the lower right region) and late apoptotic cells (located in the upper right area). Additionally, Fig 3d and Fig 4d displayed quantitative data from the annexin V assay. We discovered that the number of apoptotic cells was considerably higher in ANL cells down-regulated ARHGAP6 than in cells Negative control. These findings imply that knock down the gene ARHGAP6 increases AML cell apoptosis.

### ARHGAP6 overexpression in multiple cell lines of AML

**Fig 5:**
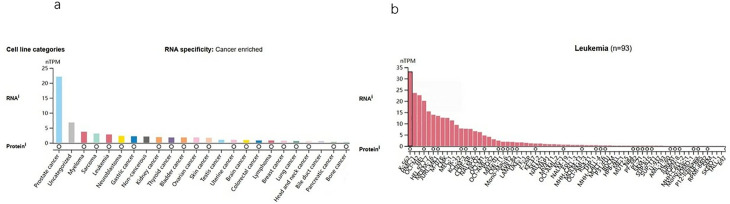
ARHGAP6 expression in cell lines. a ARHGAP6 expression in tumor cell lines, analyzed by HPA. b the expression of ARHGAP6 in leukemia Cell Lines.

In the HPA database, the expression levels of ARHGAP6 mRNA were found to be higher in several tumor cell lines (Fig 5A). We then examined ARHGAP6 expression using RNA-Seq data from cell lines listed in the HPA databases in order to clarify the importance of ARHGAP6 expression in AML cells. Similarly, ARHGAP6 has a higher expression in AML cell lines (Fig 5B).

### Mutation and DNA methylation analysis of ARHGAP6 in AML

**Fig 6:**
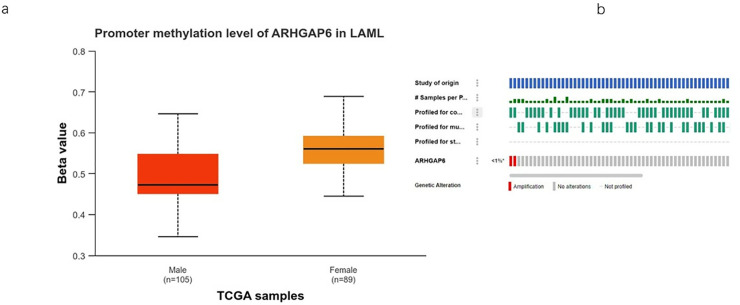
Alteration and expression of ARHGAPs in AML. a the methylation levels of ARHGAP6 are higher in female group. b Genetic alteration of ARHGAP6 in AML.

A comprehensive and easily navigable gateway website, UALCAN offers insight into TCGA gene methylation data. To determine the gene’s methylation level, we first input theARHGAP6, then chose AML data, and lastly selected methylation connections for analysis using the TCGA transcription level. The methylation levels of ARHGAP6 are significantly higher in female group (Fig 6a). The cBioPortal database (http://www.cbioportal.org) was used for mutation analysis. Amplification is the main genetic alteration in ARHGAP6 in AML (Fig 6b)

### Prognosis value of ARHGAP6 in AML

**Fig 7:**
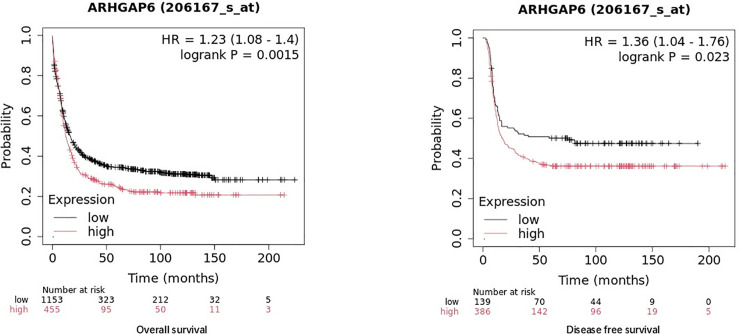
Effect of ARHGAP 6 on the survival of AML patients. a Overall survival of ARHGAP6 mRNA level in AML. b Disease free survival of ARHGAP6 mRNA level in AML.

We looked into the possibility that ARHGAP expression was related to AML patients’ prognosis. The overall survival (OS) and disease-free survival (DFS) was assessed against the expression of ARHGAP 6 using the KM plotter databases. High expression ARHGAP6 was associated poor OS and DFS in AML(p < 0.05) (Fig 7a, b).

### Functional study of relative genes

**Fig 8:**
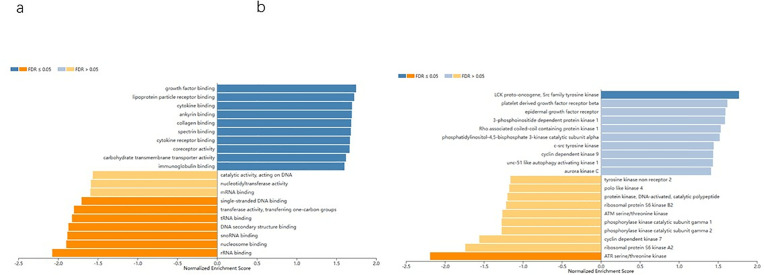
Alteration and expression of ARHGAPs in AML. a GO cellular component associated with ARHGAP6 expression b kinase target associated with ARHGAP6 genes.

Next, we investigated the potential biological role of ARHGAP6 in AML patients. The relative genes were analyzed utilizing linkedomics tools for GO cellular component and kinase target research. displays the top enriched set clusters. ARHGAP6 mainly mediated the function of growth factor binding, immunoglobulin binding, mRNA binding. Involved in LCK proto-oncogene, Src family tyrosine kinase, tyrosine kinase non receptor 2, platelet derived growth factor receptor beta and Rho associated coiled-coil containing protein kinase 1 (Fig 8a, b).

## Discussion

AML has a high degree of etiology heterogeneity and advances quickly [17]. The prognosis is still dismal because of AML relapse, even with numerous advancements in leukemogenesis pathways [18]. When examining genetic changes in AML, there are a number of crucial factors to take into account from a clinical standpoint. First, in accordance with the most recent WHO classification, it is imperative to look for genetic flaws in individuals with AML, as each mutation may define distinct pathological processes and clinical entities [19]. Since they may be employed as instruments for risk categorization, it has become more evident that certain chromosomal abnormalities and particular molecular prognostic indicators are significant. Thus, we investigate the potential biomarker ARHGAP 6 in AML through vitro study and bioinformatic tools.

The Ras superfamily includes the Rho family small GTPases, including RhoA, Rac1, and Cdc42 intracellular signaling molecules [20]. Three different protein types—Rho-selective guanine nucleotide exchange factors (RhoGEFs), GTPase-activating proteins (RhoGAPs), and guanine nucleotide dissociation inhibitors (RhoGDIs)—influence the activity of Rho GTPases [21]. RhoGAPs typically induce Rho proteins to adopt an inactive GDP-bound form, which is known as negative regulation of Rho GTPase activity [22]. A member of the RhoGAP family, ARHGAP6 (also known as RHOGAP6, RHOGAPX-1) is a protein that inhibits RhoA activity [23]. Several distinct characteristics of this novel RhoGAP were found through functional study of ARHGAP6 in cultivated cells. Transfected cells extend branching, beaded cytoplasmic processes, withdraw from the coverslip, and lose actin stress fibers. The morphological effects of ARHGAP6 expansion are not eliminated by inactivating the RhoGAP domain, unlike other RhoGAPs like p190 and Graf [24,25]. The mutation does not substantially impact process outgrowth, but it particularly impairs ARHGAP6’s capacity to counteract rhoA-mediated stress fiber production. These findings imply that ARHGAP6 has at least two actin-organizing roles, which might or might not be connected to its RhoGAP activity. Regardless of RhoA’s activation state, ARHGAP6 can also accelerate the growth of cancer. Previous research has implicated ARHGAP6 in a number of cancer biological pathways [15,26,27]. However, it is still unknown the role of ARHGAP6 in the development of AML. We demonstrate in this study that AML has significant higher expression of ARHGAP6 than control group. In AML, ARHGAP6 knockdown causes apoptosis and suppresses cell division. ARHGAP6 silencing dramatically slows the growth of AML cell lines. Through bioinformatics tools in the present study demonstrated that the methylation levels of ARHGAP6 are significantly higher in female group, Amplification is the main genetic alteration in ARHGAP6 in AML. Functional gene analysis with differentially methylation in AML shows 322 enrichments in women and 1893 in men. Survival analysis finds gender-specific epigenomic prognostic markers, with 75% dissimilarity in survival-significant gene sets between genders [28]. Both hypermethylation and hypomethylation can disrupt the cell cycle, either by silencing or activating genes that regulate it. Notably, hypomethylation significantly impacts DNA replication, potentially causing genetic disturbances and chromosomal instability.[29] We also found that ARHGAP6 mediated the function of cell activation, mRNA transcription adaptive immune response. Leukocyte proliferation. In the realm of clinical practice, despite emerging as promising anticancer agents, immune checkpoint inhibitors have exhibited limited efficacy against cancers [30]. ARHGAP6 mediated the function in growth factor binding, immunoglobulin binding, mRNA binding. Involved in LCK proto-oncogene, Src family tyrosine kinase, tyrosine kinase non receptor 2, platelet derived growth factor receptor beta and Rho associated coiled-coil containing protein kinase 1. The KM plotter datasets were used to compare the expression of ARHGAP 6 to overall survival (OS) and disease-free survival (DFS). Poor OS and DFS were linked to high expression of ARHGAP6 in AML.

This research also presents some limitations. Our findings suggest that ARHGAP6 may be a potential therapeutic target in AML, although further studies are needed to test the efficacy of specific inhibitors in preclinical and clinical settings. This study has merely confirmed the biological function of ARHGAP43 in vitro, while its in vivo function remains to be explored, which is precisely the focal point of our ongoing research. Meanwhile, the lack of patient-derived samples for some experiments and the need for validation in primary AML tissues or larger patient cohorts as key limitations of our study.

## Conclusion

From cell lines-based functional assays to bioinformatic analysis, this study presented solid evidence suggesting that ARHGAP6 s is a candidate responsible for the elevated risk of acute myeloid leukemia.

## Supporting information

S1 Fig WB image original images for blots and gels. (JPG)

S2 Fig WB image original images for blots and gels. (JPG)

S1 File Supporting data those public online tools used for building graphs. (DOCX)
