# Supplementary figures and images for: Integrated analysis of ARHGAP6 potential function and prognostic value in acute myeloid leukemia
Source: PLoS One. 2025 Oct 7;20(10):e0333409. doi: 10.1371/journal.pone.0333409 (PMC12503237; doi:10.1371/journal.pone.0333409)

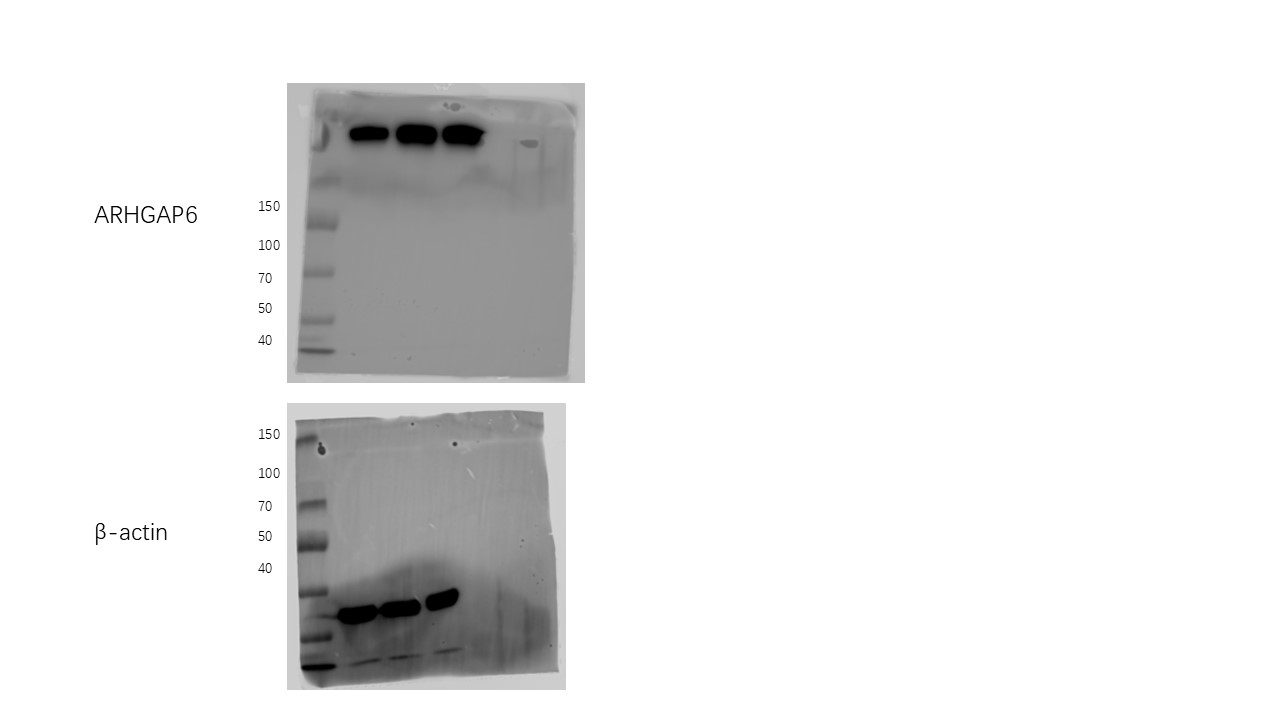

Supplement: S1 Fig — (JPG) [file pone.0333409.s001.jpg]

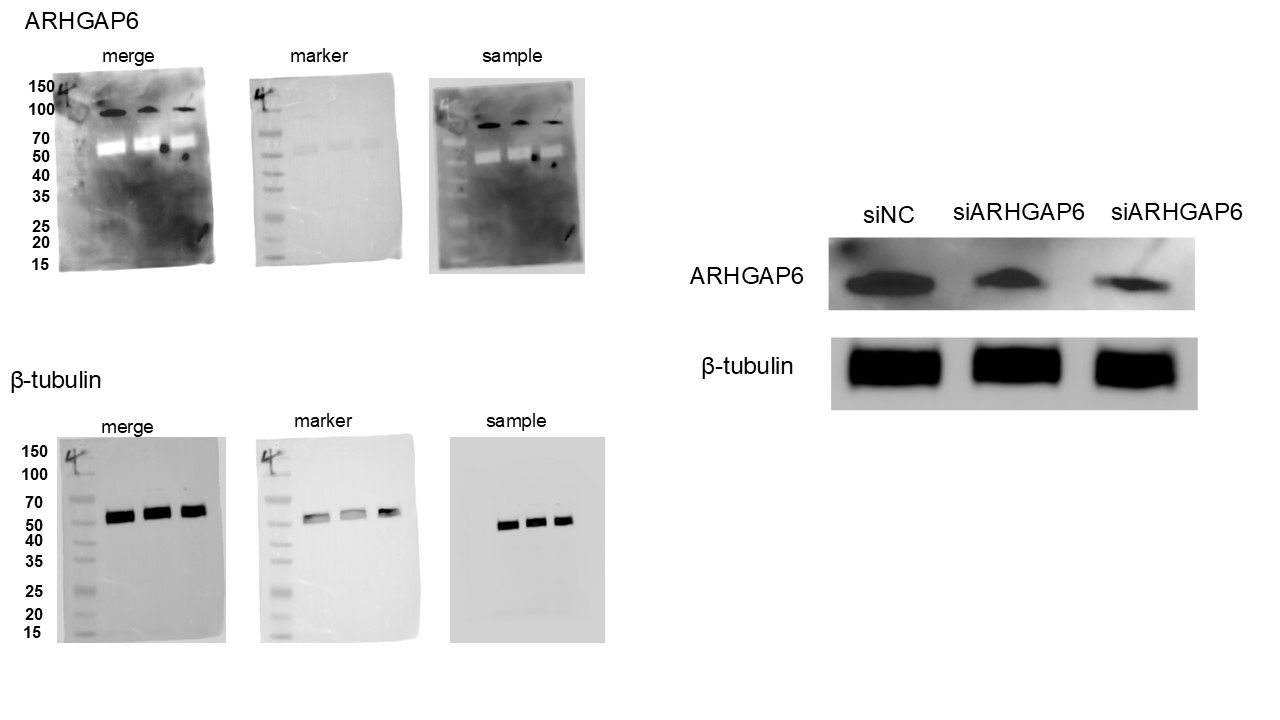

Supplement: S2 Fig — (JPG) [file pone.0333409.s002.jpg]
