# Supplementary material for: Integrated analysis of ARHGAP6 potential function and prognostic value in acute myeloid leukemia
Source: PLoS One. 2025 Oct 7;20(10):e0333409. doi: 10.1371/journal.pone.0333409 (PMC12503237; doi:10.1371/journal.pone.0333409)
Supplement: S1 File — (DOCX) [file pone.0333409.s003.docx]

Data base <https://www.proteinatlas.org/> for build graphs

Data base <https://ualcan.path.uab.edu/index.html> for build graphs

Data base <https://www.cbioportal.org/> for build graphs

Data base <https://kmplot.com/analysis/index.php?p=service> for build graphs

Data base <https://www.linkedomics.org/login.php> for build graphs

I confirm that others would be able to access all those public online tools through above websites in the same manner as the authors. I confirm that the authors did not have any special access privileges that others would not have.
